# Comparison of tissue/disease specific integrated networks using directed graphlet signatures

**DOI:** 10.1186/s12859-017-1525-z

**Published:** 2017-03-22

**Authors:** Arzu Burcak Sonmez, Tolga Can

**Affiliations:** 1 Department of Medical Informatics, Informatics Institute, Middle East Technical University, Dumlupinar Bulvari No:1, Ankara, 06800 Turkey; 2 Department of Computer Egineering, Middle East Technical University, Dumlupinar Bulvari No:1, Ankara, 06800 Turkey

**Keywords:** Integrated networks, Network comparison, Directed graphlets

## Abstract

**Background:**

Analysis of integrated genome-scale networks is a challenging problem due to heterogeneity of high-throughput data. There are several topological measures, such as graphlet counts, for characterization of biological networks.

**Results:**

In this paper, we present methods for counting small sub-graph patterns in integrated genome-scale networks which are modeled as labeled multidigraphs. We have obtained physical, regulatory, and metabolic interactions between *H. sapiens* proteins from the Pathway Commons database. The integrated network is filtered for tissue/disease specific proteins by using a large-scale human transcriptional profiling study, resulting in several tissue and disease specific sub-networks. We have applied and extended the idea of graphlet counting in undirected protein-protein interaction (PPI) networks to directed multi-labeled networks and represented each network as a vector of graphlet counts. Graphlet counts are assessed for statistical significance by comparison against a set of randomized networks. We present our results on analysis of differential graphlets between different conditions and on the utility of graphlet count vectors for clustering multiple condition specific networks.

**Conclusions:**

Our results show that there are numerous statistically significant graphlets in integrated biological networks and the graphlet signature vector can be used as an effective representation of a multi-labeled network for clustering and systems level analysis of tissue/disease specific networks.

## Background

With the accumulation of high-throughput *omics* data in public databases, integrative studies on heterogenous and dynamic biological networks have become possible. Repositories, such as Pathway Commons [1], BioGRID [2], and the Human Protein Reference Database (HPRD) [3], collect and curate associations between genes, proteins, and chemical compounds from various high and low throughput data sources. In addition, there are efforts, such as BioPAX [4], towards a standardized representation and exchange of different types of networks between databases and applications. Although the data for various types of interactions such as regulatory, metabolic, and physical interactions are available in these repositories, joint analysis of these data in a single integrated network remains a challenge. The software suite Paxtools [5] is a rich collection of methods for querying, visualizing, and converting integrated BioPAX networks; however, advanced algorithms, such as graphlet counting, are yet to be added to the expanding repository of this open source project.

In parallel with the increase in the volume of network data, modeling of the dynamic nature of networks becomes a necessity. There have been studies to obtain tissue specific protein-protein interaction networks [6, 7], functional interaction networks [8], regulatory networks [9], and pathways [10]. However, to the best of our knowledge, there is no study that provides an integrated dynamic view of physical, regulatory, and metabolic interactions.

Graphlets are small sub-graphs that provide more detailed topological statistics for a graph. As an extension of single node statistics, such as average degree and degree distribution, graphlets give a broader view around a node. Introduced by Pržulj in 2007 [11] graphlets have been shown to be effective in analysis and comparison of biological networks [12]. Due to the combinatorial expansion of different types of graphlets, computationally efficient counting of graphlets is a challenging problem. Various algorithms have been developed in recent years for counting directed or undirected graphlets of size 2–5, efficiently [13–15]. However, none of these methods consider multi-label edges. The combinatorial expansion of different types of graphlets is more dramatic when directed and multi-label edges are considered. In this paper, without tackling computational efficiency, we propose a straightforward method for counting directed multi-label graphlets of size 2–3 and assess the utility of these graphlets in tissue specific networks. To the best of our knowledge, this is the first study to address directed multi-label graphlets in integrated networks. By counting graphlets in several different tissue specific networks, we have identified many statistically significant graphlets. We also utilized graphlet signature vectors for clustering and systems level analysis of tissue/disease specific networks.

The rest of the paper is organized as follows. We first describe how we constructed tissue specific integrated networks by combining Pathway Commons networks with a human transcriptome profiling study. Next, we propose an edge encoding approach to count graphlets using simple hashing. The experimental results are followed by a brief conclusion.

## Methods

In this section, we describe the details of construction of tissue and disease specific networks using the Pathway Commons Database [1] and a human body transcriptional profiling study accessible at the NCBI GEO database [16] with accession number GSE7307. We also introduce directed and multi-labeled graphlets and outline a method for counting two to three node graphlets using a hash-based strategy. We conclude this section by describing the details of statistical significance assessment of graphlet counts.

### Datasets

We have used two main resources to acquire the data used in this study: 1) Pathway Commons [1] and 2) NCBI GEO (Gene Expression Omnibus) [16]. The details of the two data sources and how they are integrated are explained in the next two subsections.

#### Pathway Commons integrated network

Pathway Commons is an integrated resource with a repertoire of applications and data for biological pathway analysis [1]. It is also closely integrated with BioPAX [4], which is an ontological model for integration and exchange of heterogenous biological pathway data. Although the BioPAX format provides the means for integration of various pathway analysis tools, the SIF (Simple Interaction Format), originally created for use with Cytoscape [17], was sufficient for the purposes of this study. We downloaded Pathway Commons Version 8 (in extended binary SIF format) [18] for human genes identified by their HGNC [19] names. The dataset is a collection of several resources such as Reactome [20], HPRD [3], and BioGRID [2] and also contains metabolic interactions with compounds; however, in this study, we ignored compound interactions and focused only on the protein-protein associations. The resulting network contains 883,211 interactions between 19,537 human proteins. The types and numbers of interactions are given in Table 1. *interacts-with* and *in-complex-with* type interactions describe undirected associations; whereas, the other five types of interactions in the network are directed relations between proteins. All of the proteins pairs with phosphorylation and transport control relations are also indicated as having a state-change relationship; therefore, we ignored these relatively low abundance interaction types in this study. As a result, we obtained an integrated human network of 19,537 proteins with 523,498 undirected edges and 337,117 directed edges. The network contains physical, regulatory, and metabolic relations. By consolidating multiple-edges into single multi-labeled directed edges between protein pairs, the final network contains 1,521,508 directed edges between 760,754 protein pairs. In this representation, in addition to undirected interactions, each directed interaction is also modeled as a bi-directional edge such as controls-expression-of and *expression-controlled-by*. In the Methods section, we describe how multi-labeled directed edges are modeled using a binary encoding.

**Table 1:** Types and number of interactions in the Pathway Commons Version 8. The interactions are among proteins only. Metabolic interactions involving chemical compounds are ignored in this study

Interaction type	Number of gene pairs with this interaction type
Interacts-with	369,895
In-complex-with	153,603
Controls-phosphorylation-of	15,636
Catalysis-precedes	120,948
Controls-expression-of	110,013
Controls-transport-of	6960
Controls-state-change-of	106,156

#### Tissue/disease specific networks

We have used a human transcriptional profiling study for identifying genes that are expressed in specific conditions. We obtained a network for a specific tissue type by constructing the subgraph of the original Pathway Commons network using the set of active genes in that tissue. Specifically, given the integrated network as a graph **G**=(**V**,**E**) and the set of active genes for a condition as **V**_*t*_, the tissue specific graph is given as **G**_*t*_=(**V**_*t*_,**E**_*t*_) where **E**_*t*_={(*a*,*b*):∀*a*,*b*∈**V**_*t*_,(*a*,*b*)∈**E**}. The method of identifying the set of active genes in a specific condition is critical to this construction process. For this purpose, we used the results of a large scale microarray study accessible at the NCBI GEO database [16] with accession number GSE7307. Roth et al. profiled 90 distinct tissue types with several samples per tissue type (677 samples in total) using the Affymetrix U133 plus 2.0 array. Some tissues were also profiled under different states, such as disease and treated states, resulting in 141 different sample types. We have used the RMA (robust multi-array) normalized expression values to identify active genes. There are various methods for discretization of expression data [21]. In this study, we used a simple approach for identification of gene activity. We determined an approximate overall noise level, *r*, across all samples and deemed every gene with expression level above *r* as an active gene. In our experiments, we used *r*=10.0, which resulted in different networks of 11,931 nodes (retrocervical infiltrate normal sample) to 15,853 nodes (MDA-MB231 control sample) for the 141 tissue types we considered.

### Counting directed graphlets

In this section, we give the details of our graph representation approach for counting directed multi-labeled graphlets and describe how the statistical significance of graphlet counts are assessed using a set of randomized networks.

#### Edge encoding in labeled multidigraphs

Given a list of labeled interactions between pairs of proteins, we use an *adjacency-list* based implementation of the underlying graph by encoding each multi-labeled directed edge from a protein *p*_*a*_ to a protein *p*_*b*_ as an 8-bit vector. The first two bits of the edge vector from *p*_*a*_ to *p*_*b*_ is used for undirected interactions, the following three bits are used to represent directed interactions from *p*_*a*_ to *p*_*b*_, and the last three bits are used for the directed interactions from *p*_*b*_ to *p*_*a*_. Table 2 shows the assignment of labels to specific bits in the encoding.

**Table 2:** Encoding of multi-labeled directed edges as 8-bit vectors

Bit	Interaction type	Value
1	Interacts-with	0/1
2	In-complex-with	0/1
3	Catalysis-precedes	0/1
4	Controls-expression-of	0/1
5	Controls-state-change-of	0/1
6	Catalysis-succeeds	0/1
7	Expression-controlled-by	0/1
8	State-changed-by	0/1

For example, given a network with the following list of interactions between proteins *p*_*a*_ and *p*_*b*_:

*p*_*a*_ *p*_*b*_ *in-complex-with*

*p*_*a*_ *p*_*b*_ *controls-expression-of*

*p*_*b*_ *p*_*a*_ *catalysis-precedes*

The encoded edge from *p*_*a*_ to *p*_*b*_ in *p*_*a*_’s adjacency list will be 01010100; whereas, the symmetric edge in *p*_*b*_’s adjacency list will be 01100010. In this encoding, both proteins’ adjacency lists contain all the information about their interactions with their neighbors.

#### Counting algorithm

We use a hashing based strategy to count all the graphlets in a network. Our method is a naïve brute-force method, which has O(|**V**|·*d* ^*k*−1^) time complexity, where **V** is the set of nodes, *d* is the average degree in the network, and *k* is the maximum size of the counted graphlets. The size of a graphlet is the number of nodes in that graphlet. In this study, we count up to 3-node graphlets; hence, the running time complexity is O(|**V**|·*d* ^2^). This brute-force method will be prohibitively expensive for counting graphlets of larger sizes; however, in this study, we show that, in an integrated network with various types of edges, the number of distinct 3-node graphlets are on the order of thousands and 3-node graphlets are powerful in identifying different tissues and diseases.

A 2-node graphlet is simply represented by the binary encoded edge vector between two nodes. The edge vector is used as the key in a standard implementation of hash tables in the Java standard library by using the default hash function for strings. Since the edge is represented as a directed edge from a source node to a target node and the target node stores a different encoding, i.e., the symmetric encoding, of the same edge, the same 2-node graphlet is counted twice, but in two separate hash entries. These isomorphic graphlets are taken care of when we report the graphlet counts in the post-processing stage. A single representative of all isomorphic graphlet counts are reported by employing an isomorphism test before a graphlet count is reported. In the networks we analyzed, there are 56 different types of 2-node graphlets.

We represent a 3-node graphlet between three nodes *n*_*a*_, *n*_*b*_, and *n*_*c*_ as a concatenation of three binary encoded edge vectors from *n*_*a*_ to *n*_*b*_, from *n*_*b*_ to *n*_*c*_, and from *n*_*c*_ to *n*_*a*_, resulting in a string of length 24. The first two edges are enumerated from the set |**E**| using the adjacency lists and in case there is no edge between *n*_*c*_ and *n*_*a*_, the encoded edge is simply given as 00000000. Similar to counting 2-node graphlets, the concatenated encoding of length 24 is used as the key in a hash table of graphlets counts. Note that, in this appriach, a multi-label edge is used to count the most specific occurrence of a graphlet. Subsets of the labels of a multi-label edge do not contribute to the counts of more generic graphlets. We handle isomorphic graphlets in the post-processing phase and report the count for one representative graphlet. We also employ a count threshold and report only graphlets that occur at least 10 times in a network. In the networks we analyzed, there are 7346 different types of 3-node graphlets that occur at least 10 times in at least one of the 141 tissue specific networks. Counting all the 2–3 node graphlets in the largest network takes about 15 min on a MacBook Air with a 1.4 GHz Intel Core i5 processor and 4 GBs of memory.

#### Computation of statistical significance

We compute the z-score of a graphlet count by comparison against the count of that graphlet in an ensemble of randomized networks with the same degree distribution as the real networks. The degree distribution of a multi-labeled directed graph is a multi-dimensional distribution accounting for different types of incoming and outgoing edges of a node. The most frequent multi-label edge in the 141 tissue specific networks is the edge 10000000 (i.e., {*interacts-with*} edge) with an average recurrence of 542,829.58 and the least frequent multi-label edge is the edge 00101101 (i.e., {*catalysis-precedes, controls-state-change-of, catalysis-succeeds, state-changed-by*} edge) with an average recurrence of 10.83. Out of the 56 different multi-label edges, 12 of them recur more than 1000 times on the average and 28 of them recur more than 100 times on the average in the 141 tissue specific networks.

We randomized each of the 141 tissue specific networks by the edge-shuffling method. Each randomly chosen edge pair (of the same multi-label) in the graph is shuffled to replace the existing edges with a new pair of edges with the same multi-label but with different interaction partners. For example, given two directed edges from *p*_*a*_ to *p*_*b*_ and *p*_*c*_ to *p*_*d*_ with the same multi-labels, the edges *p*_*a*_→*p*_*d*_ and *p*_*c*_→*p*_*b*_ are inserted into the randomized graph. During this process, we make sure the selected edge pairs contain four distinct proteins to avoid introduction of self edges in the randomized networks. We counted each two-node and three-node graphlets in all of the 141 randomized networks and obtained the mean count, *μ*_*g*_, and the standard deviation, *σ*_*g*_, of the count of a graphlet *g*. We tested the graphlet frequency distributions in the randomized networks and verified that they follow a normal distribution. The z-score of the count *c*_*g*_ of a graphlet *g* in a tissue-specific network is then given by:

1 $$ z_{g} = \frac{c_{g} - \mu_{g}}{\sigma_{g}}  $$

If a graphlet that exists in a real tissue-specific network does not occur in any of the randomized networks, it is assigned a z-score of *∞*.

## Results

Directed multi-labeled graphlets are analyzed in three different aspects. We first investigate statistically the most significant graphlets across all tissues. Then, we assess the utility of graphlet signatures in comparison of tissue/disease specific networks. Finally, we analyze differentially occurring graphlets between two conditions.

### Statistically significant graphlets

In this section, we report the most significant graphlets in the analyzed networks. Since the graphlets with zero count in all of the randomized networks could not be assigned a numerical z-score value other than infinity, we ignored these graphlets. There are 2766 such graphlets. 275 of the remaining 4636 graphlets have z-scores over 100.00.

**Fig. 1:**
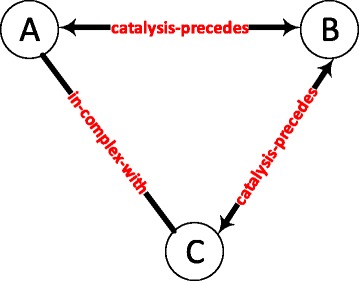
The most significant graphlet with an average z-score of 6913.36. A graphlet of three nodes. Proteins A and B play roles in different metabolic pathways by preceding each other. Proteins A and C are in the same molecular complex

Figure 1 shows the most significant graphlet with an average z-score of 6913.36 among the 141 conditions. In the 141 randomized networks, this graphlet occurs only 10.35 times; whereas, in the real networks, the graphlet occurs more than fifty thousand times on the average. The graphlet contains both metabolic and protein complex associations. In addition, a biologically interesting observation is the abundance of protein pairs that both precede and succeed each other in catalyzing a cascade of metabolic reactions.

**Fig. 2:**
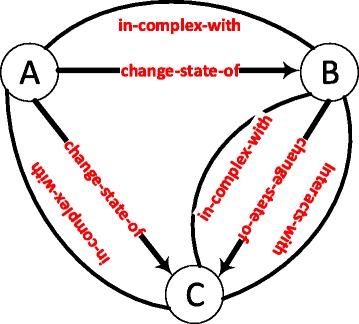
A graphlet with high z-score variation among the 141 conditions. The graphlet is a sub-complex of three proteins, in which the state change interactions exhibit a feed-forward loop motif

Due to the high abundance of statistically significant graphlets, we analyzed graphlets with high z-score variance as biologically interesting graphlets. Figure 2 shows the graphlet with largest variation of z-score among the 141 conditions. The graphlet has an average z-score of 4656.97 with a standard deviation of 1570.49. The graphlet is a sub-complex of three proteins, in which the state change interactions exhibit a feed-forward loop motif. This graphlet occurs in the myometrium disease tissue (MDT) most frequently with a z-score of 7999.00. The same graphlet occurs in the retrocervical infiltrate normal tissue (RINT) with a z-score of 1702.81 showing more than four fold decrease. This difference could be explained by the fact that the MDT network has the second highest number of active genes (15,785 nodes), while the RINT network has the least number of active genes (11,931 nodes). However, the MDT network has less number of genes than the MDA-MB231 cells control sample and there are 688 more occurrences of this graphlet in the MDT network compared to the MDA-MB231 network.

**Fig. 3:**
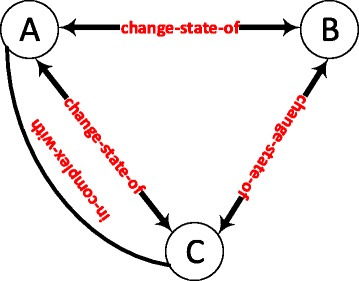
Another graphlet with high z-score variation among the 141 conditions. The graphlet is clique of three proteins in which all the members change each other’s states and two of the nodes are in a complex together

Another interesting graphlet with a high variance among the conditions is shown in Fig. 3. The graphlet is *state change* clique of three proteins in which all the members change each other’s states and two of the nodes are in a complex together. This graphlet occurs in the endometrium ovary disease tissue (EODT) network 7564 times; while, the skeletal muscle superior quadracep normal tissue (SMSQNT) tissue contains 1378 instances of this graphlet. The average occurrence of this graphlet in the randomized networks is 2.0.

### Clustering of tissues/diseases using graphlet signatures

We used the z-scores of graphlets (again by ignoring the graphlets with zero counts in the randomized networks) to represent each tissue specific network as a one-dimensional vector of graphlet signatures. There are various methods that can be applied on a set of objects represented as vectors. Below, we report our results on hierarchical clustering of tissue specific networks and principal component analysis (PCA) based reduction of graphlet signatures. We used the TM4 MeV: MultiExperiment Viewer version 4.9 [22] for conducting these analyses. MeV is originally developed for analysis of microarray data; however, the implementation of standard methods such as PCA, K-means clustering, and hierarchical clustering can be applied to any dataset with a set of objects represented as vectors.

When we performed PCA on all of the 141 graphlet signatures, the first three principle components were able to capture 96.126% of the variance in the dataset. This result shows that although there are thousands of different types of graphlets within networks, the graphlet frequencies are highly correlated and the networks can be described in a much lower dimensional space.

**Fig. 4:**
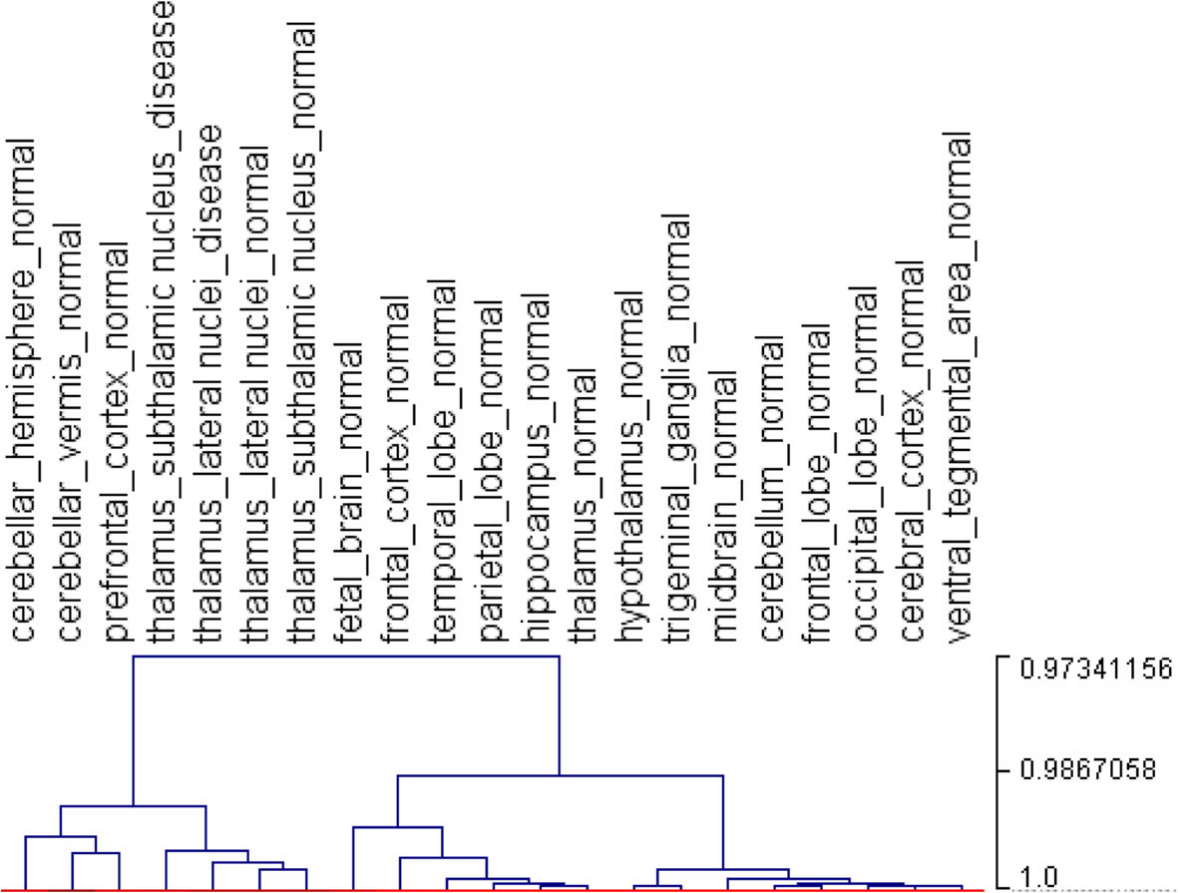
Hierarchical clustering of the brain tissue networks using graphlet signatures. Brain parts with similar functions are clustered together. Edge lengths in the hierarchical cluster show the Pearson’s correlation coefficients of graphlet signatures of different clusters

In order to show the effectiveness of clustering using graphlet signatures, we have selected a subset of 21 brain tissues. Figure 4 shows the result of hierarchical clustering of these tissues, where brain parts with similar functions are clustered together. Four of the five thalamus tissues are in the same subcluster if the tree is cut at a level to produce four main clusters. When we use the gene expression values in the microarray dataset GSE7307 to cluster the brain tissues, thalamus tissues fall in different clusters (data not shown). In addition, trivial features such as the number of tissue-specific genes do not produce the same clustering as with the graphlet signatures (data not shown). In summary, these results suggest that graphlet signatures provide a potentially different perspective on comparison of different tissues.

### Differential graphlets between two conditions

Differential counts of graphlets in the networks of disease and normal states of tissues maybe helpful in understanding disease mechanisms. We use graphlet z-score signatures to identify differentially recurring significant graphlets across networks. We conducted differential analyses on the networks of thalamus lateral nuclei, prostate gland, and skin tissue samples. The set of proteins in the differentially recurring graphlets were analyzed further for functional enrichment using DAVID functional annotation tool [23].

**Fig. 5:**
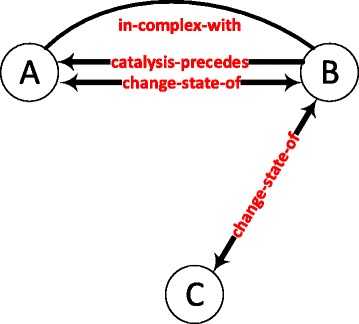
A differentially recurring graphlet in the normal and disease states of thalamus lateral nuclei tissue network. Protein A and protein B are members of the same protein complex and protein B catalyzes a reaction before protein A. All three proteins are able to change each other’s states

Figure 5 shows a differentially recurring graphlet in the normal and the disease thalamus lateral nuclei tissues with the largest fold change of z-scores. The z-score of the graphlet in the disease network is –0.08, while the z-score for this graphlet is 43.40 in the normal state network of the same tissue. We also observed a four fold difference in the counts of this graphlet in these two networks.

There are only six different proteins inducing this graphlet in the disease network and 43 different proteins induce the same graphlet in the normal state network. Four of these proteins are common in both networks. After filtering out the common proteins from the set of 43 proteins in the normal state network, the remaining proteins are assessed for functional enrichment. Table 3 shows the top three most significantly enriched functions. The count values in the table show the number of proteins in the set with that specific function. The proteins inducing this graphlet in the normal state network are related with growth factor activity and positive regulation of cell division, indicating possible loss of these functions in the disease state.

**Table 3:** Functional annotation term clusters related with the proteins of the normal thalamus lateral nuclei tissue network inducing the graphlet in Fig. 5

Annotation Cluster 1	Enrichment Score: 20.21		
Category	Term	Count	*P*-value
D GOTERM_BP_FAT	fibroblast growth factor receptor signaling pathway	13	2.3E-25
EGG_PATHWAY	hsa04010:MAPK signaling pathway	13	4.8E-12
Annotation Cluster 2	Enrichment Score: 11.04		
Category	Term	Count	*P*-value
KEGG_PATHWAY	hsa05218:Melanoma	10	5.3E-13
SP_PIR_KEYWORDS	growth factor	10	1.9E-12
GOTERM_MF_FAT	growth factor activity	10	1.2E-10
Annotation Cluster 3	Enrichment Score: 9.97		
Category	Term	Count	*P*-value
PIR_SUPERFAMILY	PIRSF001783:fibroblast growth factor	7	6.9E-14
SP_PIR_KEYWORDS	mitogen	7	8.7E-11
GOTERM_BP_FAT	positive regulation of cell division	7	4.0E-10
GOTERM_BP_FAT	regulation of cell division	7	1.3E-9

**Fig. 6:**
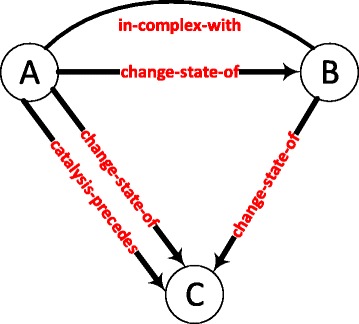
A differentially recurring graphlet in the diseased and normal states of the prostate gland network. Protein A and B are the members of the same protein complex and protein A catalyses a reaction before protein C. The *state-change* relations exhibit the feed-forward loop motif

Figure 6 shows another significant graphlet with a differential recurrence in the disease state with respect to the normal state of the prostate gland tissue. The z-score of the graphlet in the diseased prostate gland tissue network is 68.68; whereas, the z-score of the same graphlet is 7.63 in the normal network. The graphlet appears nearly nine times more in the diseased state than it does in the normal condition.

There are 74 distinct proteins that induce this graphlet in the disease network and a 28 protein subset of these proteins induce the same graphlet in the normal network. Table 4 shows the top three functional annotation clusters of the 46 proteins specific to the disease network. These proteins are highly associated with the transmembrane receptor protein tyrosine kinase signaling pathway, growth factor activity, and positive regulation of cell division.

**Table 4:** Functional annotation term clusters related with the proteins of the diseased prostate gland tissue network inducing the graphlet in Fig. 6

Annotation Cluster 1	Enrichment Score: 17.90		
Category	Term	Count	*P*-value
GOTERM_BP_FAT	TM receptor protein tyrosine kinase signaling pathway	18	5.0E-20
GOTERM_BP_FAT	enzyme linked receptor protein signaling pathway	18	6.6E-17
Annotation Cluster 2	Enrichment Score: 16.20		
Category	Term	Count	*P*-value
SP_PIR_KEYWORDS	growth factor	14	1.6E-18
GOTERM_MF_FAT	growth factor activity	14	6.0E-16
Annotation Cluster 3	Enrichment Score: 8.72		
Category	Term	Count	*P*-value
SP_PIR_KEYWORDS	mitogen	7	3.1E-10
GOTERM_BP_FAT	positive regulation of cell division	7	1.9E-9
GOTERM_BP_FAT	regulation of cell division	7	6.03E-9

**Fig. 7:**
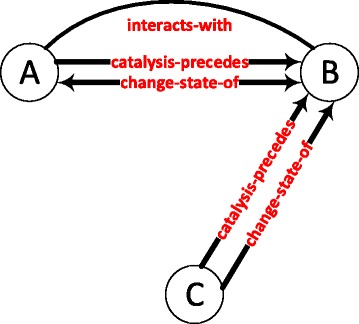
A differentially recurring graphlet in the normal and diseased state of the skin tissue network. Protein A and B interact physically and change states of each other. Protein B catalyzes a metabolic reaction before proteins A and C. Protein C also changes the state of protein B

As the last example of pair of conditions, we compare diseased and normal skin tissues. The graphlet shown in Fig. 7 occurs only in the normal state of the skin tissue and it does not occur in the disease state network at all. All the 69 proteins that induce this graphlet in the normal network are analyzed for functional enrichment. The top three most enriched term groups for the proteins that induce this graphlet is shown in Table 5. Most of the proteins participate in transmembrane transport activities and further investigation regarding the lack of these functions in the diseased skin tissue may help towards understanding the disease mechanisms.

**Table 5:** Functional annotation term clusters related with the proteins of the normal skin tissue network inducing the graphlet in Fig. 7

Annotation Cluster 1	Enrichment Score: 76.88		
Category	Term	Count	*P*-value
GOTERM_MF_FAT	ion channel activity	57	1.4E-77
GOTERM_MF_FAT	substrate specific channel activity	57	8.5E-77
GOTERM_MF_FAT	channel activity	57	6.8E-76
GOTERM_MF_FAT	passive transmembrane transporter activity	57	7.8E-76
Annotation Cluster 2	Enrichment Score: 75.69		
Category	Term	Count	*P*-value
GOTERM_MF_FAT	metal ion transmembrane transporter activity	56	1.8E-79
GOTERM_MF_ALL	cation transmembrane transporter activity	56	3.6E-68
Annotation Cluster 3	Enrichment Score: 73.73		
Category	Term	Count	*P*-value
GOTERM_BP_FAT	calcium ion transport	52	4.5E-91
GOTERM_BP_FAT	di-, tri-valent inorganic cation transport	52	2.3E-85
GOTERM_BP_FAT	metal ion transport	52	9.7E-62
GOTERM_BP_FAT	cation transport	52	9.6E-58

## Discussion

Statistically significantly recurring directed graphlets in a biological network have potential biological applications as demonstrated in the Results section. However, biological relevance of differential graphlets need to be investigated further in order to use them as practical biomarkers at the network level.

The method proposed in this paper is a brute-force counting method which is not the computationally most efficient solution for this problem. Especially, for graphlets of larges sizes, which may correspond to certain biological network modules, more efficient counting strategies should be sought. The work presented in this article is a first step that shows networks of different types can be analyzed in an integrated fashion and there are significantly recurring modules in biological networks that provide a useful characterization of genom-scale integrated networks.

## Conclusions

In this paper, we have proposed a bitwise encoding of multi-label directed edges for counting graphlets of size 2–3 with a hash-based approach. We have applied the proposed methodology on 141 tissue/disease specific networks and identified statistically significant graphlets. We have also shown that graphlets can be used for effective comparison of tissue specific networks. This study is a first attempt for counting multi-label directed graphlets in genome-scale integrated networks. In the future, computationally more efficient algorithms can be designed for counting graphlets of larger sizes. In addition, placement of graphlets in higher level systematic groups such as molecular complexes, signaling pathways, and metabolic networks will help molecular biologists interpret the results and construct novel biological hypotheses.

## Abbreviations

DAVID: The database for annotation, visualization and integrated discovery
EODT: Endometrium ovary disease tissue
GB: Giga bytes
GEO: Gene expression omnibus
GHz: Giga Hertz
HGNC: HUGO gene nomenclature committee
HPRD: Human protein reference database
MDT: Myometrium disease tissue
MeV: Multi experiment viewer
PCA: Principal component analysis
PPI: Protein-protein interaction
RINT: Retrocervical infiltrate normal tissue
RMA: Robust multi-array
SIF: Simple interaction format
SMSQNT: Skeletal muscle superior quadracep normal tissue
